# Physical Activity Patterns in Adolescents and Adults with Intellectual and Developmental Disabilities

**DOI:** 10.1249/esm.0000000000000024

**Published:** 2024

**Authors:** Brian C. Helsel, Amy E. Bodde, Lauren T. Ptomey, Joseph R. Sherman, Anna Rice, Joseph E. Donnelly, Richard A. Washburn

**Affiliations:** 1 University of Kansas Alzheimer’s Disease Research Center, Department of Neurology, The University of Kansas Medical Center, Kansas City, KS, USA; 2 Division of Physical Activity and Weight Management, Department of Internal Medicine, The University of Kansas Medical Center, Kansas City, KS, USA

**Keywords:** accelerometry, descriptive epidemiology, Down syndrome, intellectual disabilities, movement, physical activity

## Abstract

**Introduction:**

Limited information exists on the descriptive epidemiology of physical activity (PA) in individuals with intellectual disabilities (ID). The purpose of this study was to report device-measured PA and sedentary time for individuals with ID (age 10–70 yr) eligible to participate in PA promotion (i.e., self-reported PA <60–90 min·wk^−1^) and weight loss (i.e., body mass index ≥25 kg·m^−2^) clinical trials. We aimed to examine patterns of PA by diagnosis within a day and across days and US meteorological seasons.

**Methods:**

This cross-sectional study used baseline accelerometer data from individuals with ID participating in clinical trials. The Troiano adult and Freedson child cut-points were used to classify sedentary time and PA, and Wilcoxon rank sum or Kruskal–Wallis tests assessed differences by age, diagnosis, weekday versus weekend, time of day, and season. Mixed effects linear regressions explored the effect of time of day, weekend, and season on PA and sedentary time.

**Results:**

There were 330 individuals (57% female) who had valid wear time with an average of 14 ± 18 min·d^−1^ of moderate-to-vigorous PA (age 10–17 yr: 13 ± 16 min·d^−1^; age 18–24 yr: 18 ± 23 min·d^−1^; age ≥25 yr: 12 ± 13 min·d^−1^). Fewer minutes of moderate-to-vigorous PA were observed on the weekends (*β* = −0.10, *P* < 0.001) and in the morning (before 0900 h, *β* = −0.23, *P* < 0.001) and evening (after 1900 h, *β* = −0.32, *P* < 0.001) compared to weekdays and late afternoon (1500–1659h).

**Conclusion:**

The promotion of PA to individuals with ID is needed, particularly during times when these individuals are not in school or at work (i.e., mornings, evenings, and weekends). Future research should explore PA patterns in adolescents and adults with ID who are normal weight and regularly participating in PA, sport, and fitness programs.

## INTRODUCTION

Individuals with intellectual disabilities (ID) have low levels of moderate-to-vigorous physical activity (MVPA) compared to their typically developing peers (1,2). IDs are defined as disabilities originating before 22 yr of age characterized by significant limitations in both intellectual functioning (intelligence quotient (IQ) <75) and adaptive behaviors (3). IDs are present in all individuals with Down syndrome (DS) and one-third of individuals with Autism diagnoses (4), and result from a variety of additional etiologies including fetal alcohol syndrome, birth defects, and trauma (3,5). Current physical activity (PA) guidelines from the US Department of Health and Human Services recommend 150 and 420 min·wk^−1^ of MVPA for adults and adolescents, respectively (6). However, it is estimated that <10% of adults with ID (compared to ~47% of typically developed adults) and ~6% of adolescents with ID (compared to ~25%–29% of typically developing adolescents) meet the MVPA recommendations (7-10).

Low MVPA is associated with decreased cardiovascular fitness (11,12), a higher prevalence of obesity, and an increased risk for chronic disease in individuals with ID (13,14). Additionally, an attenuated physical capacity may impair performance of activities of daily living and limit vocational capability hindering the ability of persons with ID to work and live independently (15,16). Prior research suggests a decrease in MVPA from childhood to adulthood as school-related MVPA and active play decrease (2,17). Challenges in intellectual function, behavior problems, financial limitations or the need for transportation, adaptations, and supports may limit participation in traditional MVPA opportunities in adulthood (e.g., team sports, active transport to school or work, fitness centers) (18,19). Furthermore, PA participation may differ between individuals with ID based on their primary diagnoses. For example, Phillips and Holland (20) found that device-measured PA was significantly lower in individuals with DS compared to other IDs. Individuals with DS face physiological constraints affecting PA participation including gait abnormalities, hypotonia, and congenital heart defects that may make PA difficult and may be further compounded by a lack of support, interest, or accessible PA programming (21-23).

Information on the descriptive epidemiology of PA and sedentary behavior is important for designing interventions tailored to individuals with ID. Studies comparing PA and sedentary behavior among individuals with ID using portable accelerometers often report the average daily or weekly minutes of MVPA to assess group differences or facilitate a comparison to PA guidelines (6,17,24-26). To improve upon prior research, we present an intraday analysis of device-measured PA and sedentary time collected at baseline from over 300 persons with ID (age 10–70 yr) enrolled in four clinical trials conducted by our group (27-30). Herein, we describe PA and sedentary time by age, sex, and ID diagnosis (DS vs non-DS) and variations in these outcomes within a day, across days (weekday vs weekend day), and by US meteorological season (winter, spring, summer, and fall). We hypothesize that PA will be lowest in adults with DS and that structured days (e.g., weekdays, school days) will yield higher PA estimates compared to nonstructured days (31).

## METHODS

### Participants

This is a secondary analysis of baseline data from four clinical trials in individuals with ID conducted by our research group that used similar inclusion criteria (27-30). Inclusion criteria common to all four trials included residential status (i.e., living at home with a parent/guardian or in a supported living environment with a caregiver who agreed to serve as a study partner), Internet access in the home, ability to participate in MVPA with physician clearance indicating no contraindications for exercise, and the capability to understand directions as well as communicate preferences, wants, and needs through spoken language. Two trials required participants to be overweight or obese (27,30), and one trial required a DS diagnosis without dementia (28). Participants with chronic health conditions, pregnancy, or recent weight loss (27,30), or those participating in a regular exercise routine (*n* = 14 adults self-reported PA <60 min·wk^−1^ (28), *n* = 5 adolescents self-reported PA <90 min·wk^−1^ (29)) were excluded. The studies were approved by the University of Kansas Medical Center’s Human Research Protection Program (approval numbers: 00143836, 00001857, 00140653, and 00140784), and informed consent or informed consent and assent were obtained from participants and their parents or caregivers.

### Outcomes

Data for this analysis were collected in the United States between October 2015 and November 2022. Trained staff collected demographics (e.g., age, sex, race/ethnicity, and diagnosis) and study-specific measures before providing the persons with ID a portable ActiGraph wGT3X-BT accelerometer (ActiGraph Corp, Pensacola, FL, USA) to assess free-living PA and sedentary time. Participants were asked to wear the accelerometer on a belt at the anterior axillary line of their nondominant hip over 7 consecutive days. The research team provided instruction on the correct positioning and orientation of the accelerometer and a prestamped, addressed envelope to return the accelerometer the day after the programmed end date. Data were collected at 60 Hz and aggregated over 60-s epochs; nonwear time was defined as at least 90 consecutive minutes of zero counts with a 1- to 2-min allowance of counts between 0 and 100 (32). A minimum wear time of three 8-h days, including a weekend day, was required for inclusion in the analysis. These wear time criteria were selected to be more inclusionary of individuals with ID who may be less compliant with physical behavior tracking compared to those without ID. Sedentary time and PA were determined using the vertical axis Troiano adult (sedentary 0–100 counts per minute, light PA 101–2019 counts per minute, and MVPA ≥2020 counts per minute (33)) or Freedson age-specific child cut-points (34,35). Sedentary time and PA, including activity counts, were summed to get per hour and weekly estimates of sedentary time and PA.

### Statistical Analysis

Means and standard deviations (SD) are reported for continuous measures and frequencies and percentages are used to represent categorical variables. We used Wilcoxon rank sum tests to assess differences in activity counts; percent of valid wear time spent in sedentary behavior, light activity, and MVPA; and weekend behavior by ID diagnosis (i.e., with and without DS) and sex for each age category (i.e., adolescents 10–17 yr, young adults 18–24 yr, and adults ≥25 yr), reflective of common developmental categories (36,37). Additionally, we used Kruskal–Wallis tests to evaluate differences in MVPA by season and time of day among the different ID diagnoses and age categories. Mixed effects linear regressions were used to determine the effect of time of day, weekend, and US meteorological season on activity counts and minutes of sedentary time, light PA, and MVPA per hour adjusting for age, sex, diagnosis, year of assessment, and wear time. Mixed effects linear regressions were conducted using the lme4 package in R (version 4.1.2; R Foundation for Statistical Computing, Vienna, Austria) with random intercepts varying among the participants and days of observation within each participant. Finally, we calculated the number and percentage of persons with ID by diagnosis and age category who achieved the MVPA recommendations of 150 min·wk^−1^ for adults and 420 min·wk^−1^ for adolescents (6). Accelerometer processing and analysis were completed using R.

## RESULTS

Most (330/388; 85%) of the individuals with ID (age 22 ± 10 yr, 57% female, 83% White, 58% Down syndrome) had valid accelerometer data (Table 1). Nearly all of the individuals in our sample with valid accelerometer data had 7 valid days (325/330; 98%). There were 123 adolescents between the ages of 10 and 17 yr (age 14 ± 2 yr, 49% female, 80% White), 112 young adults between the ages of 18 and 24 yr (age 20 ± 2 yr, 62% female, 84% White), and 95 adults 25 yr of age and older (age 35 ± 9 yr, 60% female, 84% White).

**Figure 1.**
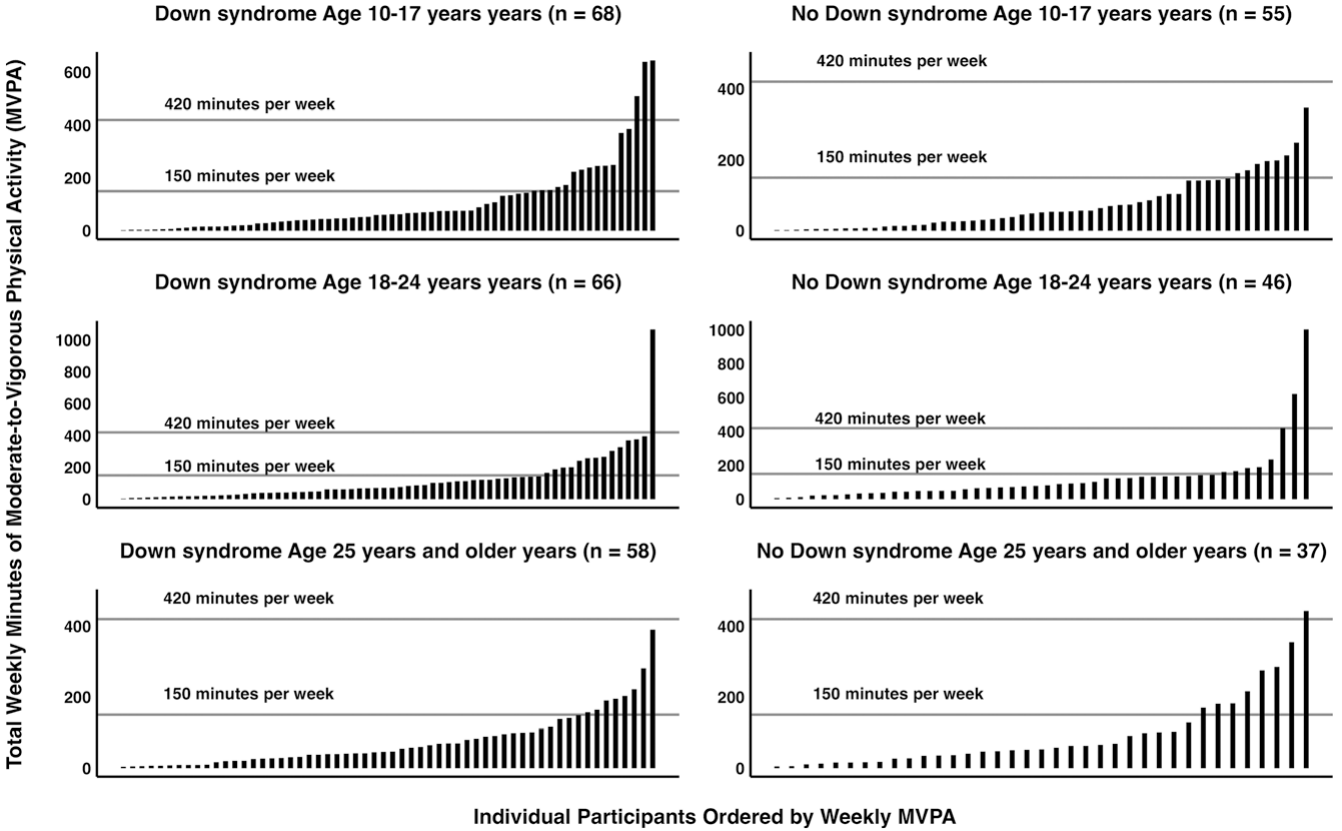
Individual variability in minutes per week of MVPA by age category for individuals with and without Down syndrome.

Average MVPA was 13.2 ± 16.3 min·d^−1^ in adolescents, 17.6 ± 22.8 min·d^−1^ in young adults, and 12.0 ± 12.7 min·d^−1^ in adults. Figure 1 shows the individual variability in minutes per week of MVPA by ID diagnosis across age categories in a column chart ordered from the lowest to highest values. Only 41 of the 330 (12.4%) persons with ID met the MVPA recommendations of 150 min·wk^−1^ (DS: *n* = 22, 17.7%; non-DS: *n* = 16, 19.3%) and 420 min·wk^−1^ (DS: *n* = 3, 4.4%; non-DS: *n* = 0) for adults and adolescents, respectively.

**Figure 2.**
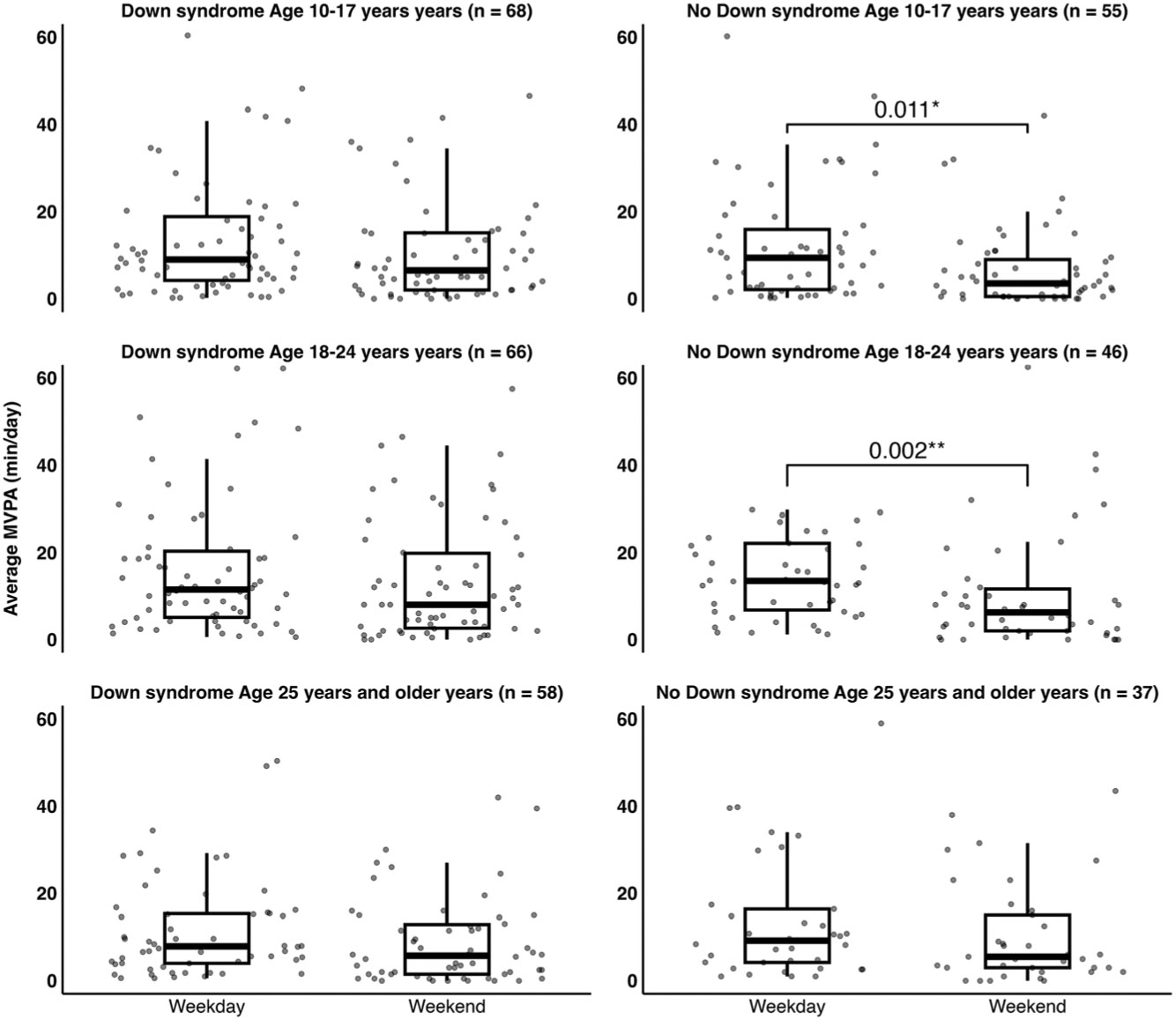
Average moderate-to-vigorous physical activity (MVPA; minutes per day) by weekend across age categories for individuals with and without Down syndrome. *P < 0.05. **P < 0.01.

Wilcoxon rank sum tests for device-measured sedentary time and PA are presented in Table 2. Sedentary time, light PA, and MVPA are represented in minutes per valid day. We found that adolescents 10–17 yr old with DS had more activity counts (*P* = 0.048) and light PA (*P* = 0.002) than adolescents without DS. Additionally, female young adults 18–24 yr old accumulated more minutes per day of MVPA than male young adults (*P* = 0.046). We also observed 5.2 (*P* = 0.01) and 7.7 (*P* = 0.002) fewer minutes per day of MVPA on the weekend for adolescents and young adults without DS, respectively (Fig. 2).

**Figure 3.**
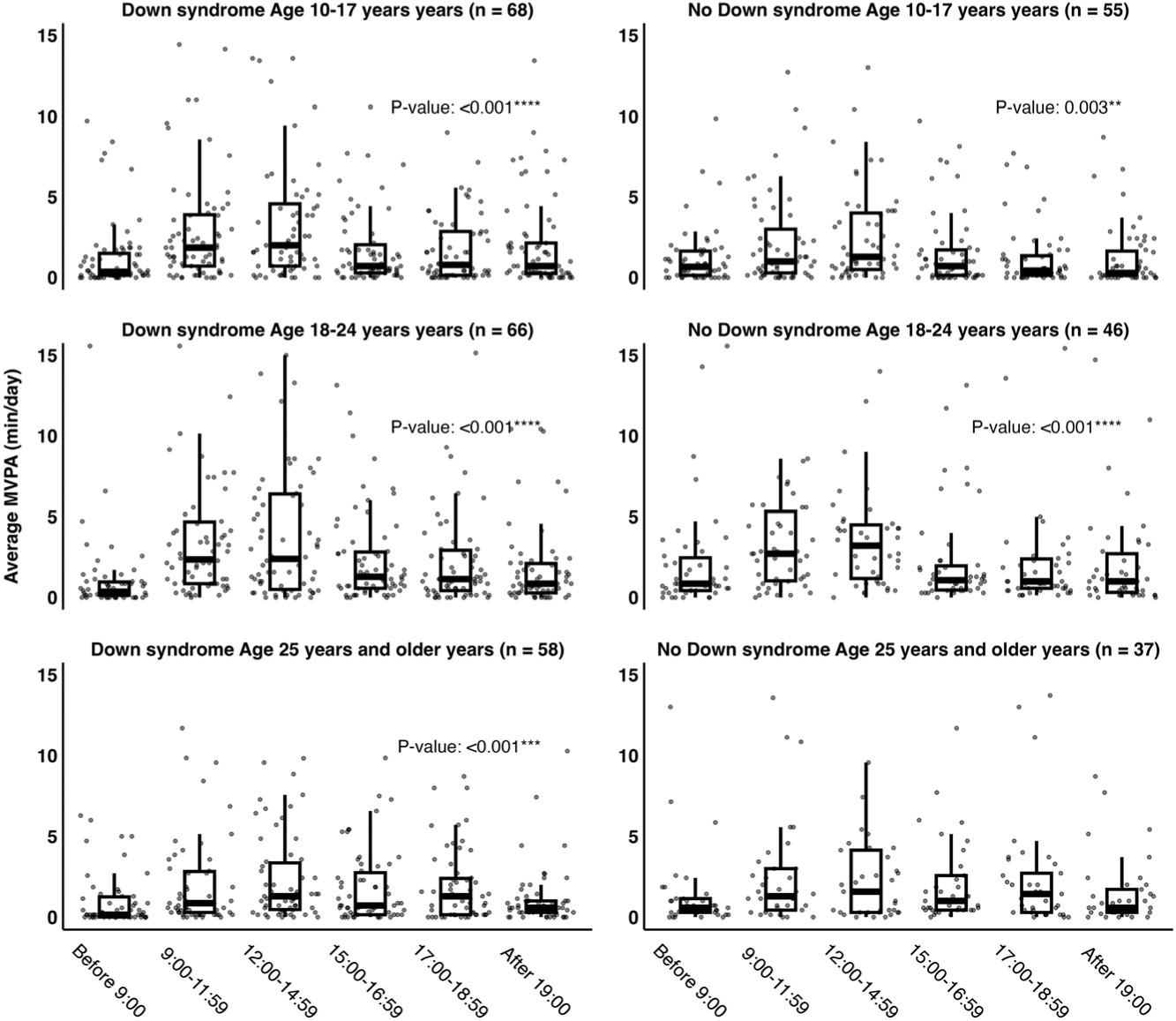
Average moderate-to-vigorous physical activity (MVPA; minutes per day) by time of day across age categories for individuals with and without Down syndrome. **P < 0.01. ***P < 0.001. ****P < 0.0001.

Average MVPA by 24-h time category (i.e., before 0900, 0900–1159, 1200–1459, 1500–1659, 1700–1859, and after 1900 h) is presented in Figure 3. The time categories were selected to segment the day into intervals that captured PA before, during, and after the school or workday (38,39). We found that more MVPA was accumulated between 0900 and 1459 h among the different age and diagnostic groups with significant differences among the time categories in all groups except for adults without DS (age ≥25 yr). We did not find any seasonal differences among the diagnosis and age categories.

The results from the mixed effects linear regressions are presented in Table 3. We found significantly lower light PA (*β* = −0.35, *P* = 0.003), MVPA (*β* = −0.10, *P* < 0.001), and activity counts (*β* = −758, *P* < 0.001) and higher sedentary time (*β* = 0.45, *P* < 0.001) on weekends compared to weekdays. We also discovered that participants accumulated more activity counts (*β* = 1240, *P* = 0.04) and were less sedentary (*β* = −1.02, *P* = 0.03) during the summer compared to the winter. MVPA was lower in the morning (before 0900 h; *β* = −0.23, *P* < 0.001) and evening (after 1900 h; *β* = −0.32, *P* < 0.001) compared to MVPA in the later afternoon (1500–1659 h, reference group). Similarly, we observed significantly lower light PA and activity counts during these time categories and significantly higher sedentary time. Persons with DS participated in more minutes per hour of light PA (*β* = 1.26, *P* < 0.001) and fewer minutes per hour of sedentary time (*β* = −1.25, *P* = 0.003) than individuals without DS, but we did not see any differences in MVPA by diagnosis. Additionally, adults aged ≥25 yr participated in fewer minutes per hour of MVPA (*β* = −0.27, *P* = 0.009) compared to young adults (18–24 yr old).

## DISCUSSION

Individuals with ID had low MVPA (14.3 ± 18.0 min·d^−1^), with only 12.4% of our sample meeting the MVPA recommendations (6), including 2.4% (*n* = 3) of adolescents and 18.4% (*n* = 38) of adults. However, the percentage of individuals with ID meeting the MVPA recommendations should be interpreted within the context of the sample; participants were recruited for PA and weight loss studies and were less active than those already participating in regular physical activities (e.g., Special Olympics athletes). Nevertheless, this is consistent with a study by Stanish et al. (8) and a systematic review by Dairo et al. (7) that found that only 6% of youth and 9% of adults with ID met the PA recommendations, respectively. Other studies using device-based measures of PA found similar MVPA levels in individuals with ID. For example, Melville et al. (40) reported that 54 adults with ID at baseline of a weight loss intervention spent 13.1 ± 16.2 min·d^−1^ in MVPA or 2.0% ± 2.7% of the time that they were monitored. These researchers employed similar data processing methods to our study and arrived at a nearly identical estimation of MVPA. However, Phillips and Holland (20) reported a much higher estimate of MVPA for individuals with DS (29.8 ± 15.6 min·d^−1^) compared to those with other ID (41.6 ± 23.0 min·d^−1^) possibly suggesting different PA levels than those who are recruited for a weight loss intervention (i.e., BMI ≥30 kg·m^−2^). Similarly, Case et al. (41) found a higher percentage of youth with ID meeting the 60 min·d^−1^ PA recommendation (19%) compared to our study (4.4%); however, they used parent-reported PA rather than device-measured PA. Nevertheless, our findings suggest that fewer adolescents and adults with ID meet the PA recommendations compared to the ~25%–29% of adolescents and ~47% of adults in the general US population (10,42).

Factors such as younger age and male sex have previously been found to be independent predictors of achieving PA guidelines both in adults with ID and the general population (7,10,17,20,43). We observed fewer accumulated minutes per hour of MVPA for adults (≥25 yr old) compared to young adults (18–24 yr old) and a slightly higher percentage of wear time spent in MVPA for males compared to females. The finding that young adult men had the highest percentage of wear time spent in MVPA is consistent with a previous systematic review and meta-analysis that found women with ID to be less active than men (24). Nevertheless, the overall low levels of MVPA suggest a need for PA promotion strategies that suit the preferences and interests of both sexes and address the common social barriers to PA, including the acceptance of inactive lifestyles, lack of adapted or inclusive programs, transportation difficulties, and a lack of caregiver support for PA (44).

Adolescents with DS participated in a higher percentage of light activity (*P* = 0.004) and were less sedentary (*P* = 0.007) than adolescents without DS. Additionally, adolescents with DS acquired ~43,000 more activity counts (*P* = 0.048) than adolescents without DS, suggesting a greater amount of total movement. However, no differences by diagnosis were observed in minutes per day of MVPA across age categories. Our findings contrast with Melville et al. (40) and Phillips and Holland (20), who found that individuals with DS had reduced odds for meeting PA recommendations and participated in fewer minutes of PA than individuals with other ID. Comparing adults with DS to those with Williams syndrome and Prader–Willi syndrome, Nordstrøm et al. (45) found no differences in MVPA between the three groups. Nevertheless, individuals with DS may face additional barriers to PA participation including gait abnormalities, hypotonia, overweight and obesity, and congenital heart defects that may make PA difficult (21). Future interventions may consider targeted strategies specific to the needs of individuals with DS such as providing proper foot-wear, incorporating acclimatization periods, identifying PA preferences, and making programs that are accessible, supported, and adapted to the needs and abilities of individuals with DS (46,47).

Our findings that MVPA is lower before 0900 h, after 1900 h, and on the weekend across all age categories is consistent with the structured day hypothesis in children who are typically developing (31,48-51). The structured day hypothesis suggests that children’s PA behavior may be higher on days that contain more structure (i.e., preplanned activities in an adult-supported environment) such as school days, which may continue until an individual with ID reaches 21 yr of age (31,52,53). Similar to our findings, Queralt et al. (17) found that adolescents with mild to moderate ID had lower daily PA levels on weekend days. Additionally, school-based PA in the study by Queralt et al. (17) represented ~50% of daily PA, suggesting that physical education classes, recess, and classroom activities could be appropriate mechanisms for promoting PA in youth with ID (54,55). The structured day hypothesis has not been proposed for adults, but previous studies suggest that adults with ID are less active during the evening and on the weekends (56,57). These previous findings, along with our current analysis, suggest that community-based PA programs and interventions in the morning, in the evenings, and on weekends should be considered to develop strategies that will increase the number of persons with ID achieving PA levels associated with health benefits.

Seasonal differences in MVPA participation have been observed in typically developed adolescents and adults. For example, a large (*n* = 10,918) longitudinal study found that adolescents were twice as likely not to meet MVPA recommendations in the winter compared to summer (58), and a scoping review of 110 studies demonstrating that MVPA participation is consistently lower in the winter compared to summer (59). Individuals with ID in our study participated in fewer minutes of sedentary time and more light activity during the summer compared to the winter. However, there was no seasonal effect on MVPA in our study. These results are consistent with a study by Zheng et al. (60), who found a positive correlation between sedentary time and lower temperatures. In contrast, a study by Sit et al. (61) reported that children with disabilities living in Hong Kong engaged in more MVPA at school during winter (18.6 min·d^−1^) compared to summer (15.6 min·d^−1^) months possibly due to higher temperatures and relative humidity during the summer.

Strengths of this study include the intraday comparisons of data collected from portable accelerometers in a large sample of individuals with ID. The 330 participants with valid accelerometer data in the current study are the largest sample to date, with prior studies including between 8 and 175 individuals with ID (62,63). Limitations include the use of a cross-sectional study design precluding causal inference; inconsistent inclusion and exclusion criteria that included participation in a regular exercise routine as an exclusionary criterion for two trials (28,29); the inability to generalize the results to geographical areas with various weather patterns, racial and ethnic groups other than non-Hispanic White, and more active individuals with ID participating in sport and fitness activities (e.g., Special Olympics); and poor compliance with our accelerometer protocol for the assessment of MVPA with 58 individuals being excluded from the analysis because of nonvalid wear time.

The results of our study suggest that only 12.4% of persons with ID achieve MVPA recommendations. We found that average MVPA was generally low (14.3 ± 18.0 min·d^−1^) with no differences between those with and without DS across different age categories. Adults ≥25 yr old engaged in fewer minutes per hour of MVPA than young adults (age 18–24 yr) and individuals with ID achieved fewer minutes per hour of MVPA during nonstructured time periods (i.e., before 0900 h, after 1900 h, and on weekends). The results suggest that programs promoting PA to persons with ID are needed, especially during days and times containing less structure (i.e., mornings, evenings, and weekends).

## Figures and Tables

**Table 1:** Sample Characteristics and Physical Activity by Age Category.

	Overall (*n* = 330)	10–17 yr (*n* = 123)	18–24 yr (*n* = 112)	≥25 yr (*n* = 95)	*P*
Age	22.1 ± 9.7	14.4 ± 2.0	20.0 ± 1.8	34.7 ± 8.8	
Female	187 (57%)	60 (49%)	70 (62%)	57 (60%)	
White	273 (83%)	99 (80%)	94 (84%)	80 (84%)	
Not Hispanic or Latino	306 (93%)	116 (94%)	99 (88%)	91 (96%)	
Down syndrome	192 (58%)	68 (55%)	66 (59%)	58 (61%)	
US meteorological season					
Winter	99 (30%)	41 (33%)	36 (32%)	22 (23%)	
Spring	85 (26%)	38 (31%)	23 (21%)	24 (25%)	
Summer	66 (20%)	27 (22%)	19 (17%)	20 (21%)	
Fall	80 (24%)	17 (14%)	34 (30%)	29 (31%)	
Activity					
Wear	694 ± 178	693 ± 199	683 ± 151	708 ± 178	0.48
Activity counts*^a^*	198.4 ± 113.0	223.9 ± 112.5	192.7 ± 127.4	172.2 ± 86.5	<0.001
Sedentary	436.2 ± 147.4	426.9 ± 159.7	434.1 ± 132.9	450.8 ± 147.6	0.28
Light	243.2 ± 88.0	252.3 ± 90.5	231.4 ± 86.8	245.1 ± 85.2	0.08
MVPA	14.3 ± 18.0	13.2 ± 16.3	17.6 ± 22.8	12.0 ± 12.7	0.02

Data presented as mean ± standard deviation or *n* (%). Values calculated via Kruskal–Wallis rank sum test. MVPA, moderate-to-vigorous physical activity.

a Activity counts are reported in thousands of counts.

**Table 2:** Differences in Device-based Measures of Physical Activity for Individuals with Intellectual Disabilities.

Variable	Down Syndrome	No Down Syndrome	*P*	Female	Male	*P*
**Age 10–17 yr, *n***	**68**	**55**		**60**	**63**	
Wear time	714 ± 183	667 ± 215	0.20	703 ± 184	683 ± 213	0.30
Counts	243 ± 120	200 ± 99	0.048	228 ± 104	220 ± 121	0.50
Sedentary	422 ± 142	433 ± 181	0.80	421 ± 151	432 ± 169	0.80
Light	276 ± 91	223 ± 82	0.002	268 ± 95	237 ± 84	0.11
MVPA	15 ± 19	10 ± 11	0.20	13 ± 16	14 ± 17	0.50
**Age 18–24 yr, **n****	**66**	**46**		**70**	**42**	
Wear time	679 ± 145	689 ± 162	0.90	696 ± 155	662 ± 144	0.40
Counts	200 ± 136	183 ± 115	0.70	189 ± 144	198 ± 94	0.12
Sedentary	422 ± 119	452 ± 151	0.30	454 ± 136	401 ± 123	0.07
Light	240 ± 93	219 ± 76	0.30	224 ± 78	244 ± 100	0.40
MVPA	17 ± 22	18 ± 24	0.70	18 ± 27	17 ± 12	0.046
**Age 25 yr and older, *n***	**58**	**37**		**57**	**38**	
Wear time	729 ± 171	676 ± 188	0.20	710 ± 178	705 ± 181	>0.90
Counts	174 ± 80	170 ± 97	0.60	162 ± 75	187 ± 101	0.40
Sedentary	459 ± 150	439 ± 145	0.70	454 ± 153	446 ± 140	>0.90
Light	259 ± 82	224 ± 87	0.09	246 ± 85	244 ± 86	0.80
MVPA	11 ± 11	14 ± 15	0.50	10 ± 10	15 ± 15	0.20

Values presented as mean ± standard deviation unless otherwise noted. Values calculated via Wilcoxon rank sum tests. Activity counts are presented in thousands of counts. Wear time, sedentary, light, and moderate-to-vigorous physical activity (MVPA) are presented as minutes per valid day.

**Table 3:** The Impact of the Season, Weekend, and Time of Day on Sedentary Time and Physical Activity (Minutes per Hour) in Individuals with Intellectual Disabilities Using Mixed Effects Linear Regression.

	Sedentary Time			Light Activity			MVPA			Activity Counts		
	*β*	95% CI	*P*	*β*	95% CI	*P*	*β*	95% CI	*P*	*β*	95% CI	*P*
Gender: female	0.19	−0.54 to 0.91	0.61	−0.10	−0.76 to 0.57	0.77	−0.09	−0.25 to 0.08	0.30	−559.8	−1510.2 to 390.6	0.25
Down syndrome	−1.25	−2.06 to −0.44	0.003	1.26	0.52 to 2.00	<0.001	−0.01	−0.19 to 0.17	0.89	590.7	−469.8 to 1651.2	0.27
Weekend	0.45	0.21 to 0.69	<0.001	−0.35	−0.58 to −0.12	0.003	−0.10	−0.15 to −0.05	<0.001	−757.9	−1068.9 to −446.8	<0.001
Age category												
Age 10–17 yr	−0.44	−1.32 to 0.43	0.32	0.64	−0.16 to 1.44	0.12	−0.19	−0.39 to 0.00	0.051	1107.1	−38.2 to 2252.5	0.06
Age 18–24 yr	—	—	—	—	—	—	—	—	—	—	—	—
Age ≥25 yr	0.14	−0.76 to 1.04	0.76	0.13	−0.70 to 0.96	0.76	−0.26	−0.47 to −0.06	0.01	−1223.1	−2406.1 to −40.1	0.04
Season												
Spring	−0.35	−1.18 to 0.48	0.41	0.21	−0.56 to 0.98	0.60	0.15	−0.04 to 0.33	0.11	555.7	−527.4 to 1638.8	0.31
Summer	−1.02	−1.93 to −0.10	0.03	0.83	−0.01 to 1.68	0.054	0.18	−0.02 to 0.38	0.08	1240.1	42.3 to 2438.0	0.04
Fall	−0.34	−1.29 to 0.62	0.49	0.23	−0.65 to 1.11	0.60	0.11	−0.10 to 0.32	0.31	677.3	−568.9 to 1923.5	0.29
Winter	—	—	—	—	—	—	—	—	—	—	—	—
Time category												
Before 0900 h	2.05	1.74 to 2.36	<0.001	−1.84	−2.12 to −1.55	<0.001	−0.23	−0.32 to −0.14	<0.001	−2598.7	−3103.0 to −2094.4	<0.001
0900–1159 h	−0.68	−1.00 to −0.36	<0.001	0.60	0.30 to 0.89	<0.001	0.07	−0.02 to 0.17	0.12	227.7	−299.7 to 755.1	0.40
1200–1459 h	−0.79	−1.10 to −0.47	<0.001	0.71	0.42 to 1.01	<0.001	0.06	−0.03 to 0.15	0.21	261.4	−265.1 to 787.8	0.33
1500–1659 h	—	—		—	—		—	—		—	—	
1700–1859 h	0.34	−0.01 to 0.69	0.06	−0.38	−0.71 to −0.06	0.02	0.04	−0.07 to 0.14	0.49	−380.6	−957.4 to 196.2	0.20
After 1900 h	2.49	2.18 to 2.79	<0.001	−2.18	−2.47 to −1.90	<0.001	−0.32	−0.41 to −0.23	<0.001	−3132.3	−3634.8 to −2629.7	<0.001

*P* values were estimated using the Satterthwaite approximation. Mixed effects linear regressions were adjusted for wear time and year of assessment.

CI, confidence interval; MVPA, moderate-to-vigorous physical activity.

## Data Availability

The dataset(s) generated and/or analyzed during the current study is available from the corresponding author upon reasonable request.
